# On the Pathology of Deposits of Urates

**Published:** 1865-02

**Authors:** George Harley

**Affiliations:** Professor in University College, and Assistant-Physician to University College Hospital


### ON THE PATHOLOGY OF DEPOSITS OF URATES.

By DR. GEORGE HARLEY,
Professor in University College, and Assistant-Physician to University College Hospital.

Deposits of Urates are of a yellow or pink color, according to the amount and kind of urohæmatin present in the liquid. The higher the color the more febrile the state of the body. At one time their presence in the urine was regarded as a sign of the crisis of disease, and hence they received the name of critical discharges. Modern investigation has, however, proved that they can not always be looked upon in this light, as they may also appear under a variety of conditions, which, although abnormal in themselves, are not truly diseased states, according to the usual meaning of the word. Thus, for example, urates may be deposited in the urine after a slight attack of indigestion, the result of over eating or drinking. Great exertion, especially if accompanied by profuse sweating will also cause them to appear. Hence we find them present in the urine after a fatiguing walk, a long day's hunting, or even after a ball, or other such occasional amusement, especially if it has been associated with much mental excitement. Hard study, and even a fright, will in some persons be followed by a deposit of urate of soda. A sudden change in the mode of life is a very common cause of their appearance, as, for example, confinement in town to country people, or a few days' residence in France to an Englishman unaccustomed to French dishes. Under none of these circumstances can the deposit be said to indicate a state of disease. It does nothing more than denote a temporary abnormal condition of the system which may soon disappear without treatment. If, however, the deposit, instead of being merely temporary, lasts for some days, that of itself would denote that something more than a mere ephemeral derangement of the system is present, and may deserve immediate attention. A deposit in the urine is always a sign of something being wrong, and although, as we have seen, it may occur from very trivial causes, whenever it takes place without appreciable cause, in the otherwise apparently healthy, it is a sign not to be disregarded, as, under such circumstances, it is not unfrequently either the forerunner or associate of gravel or stone. Uric acid in some form or other is the commonest ingredient of all calculi, and there is no period of life exempt from them.

Urates are a very common deposit in the course of acute disease, and they even seldom fail to recur at some period or other in the course of chronic affections. It is, however, only in diseases of an acute, febrile or inflammatory type that their sudden appearance can be regarded as indicative of a crisis. Their sudden appearance is due to an important change having occurred in the condition of the patient, and in general, though not always, it is a change for the better. Such, for example, is observed to occur in cases of gout and rheumatism where the climax has been reached. So also in pneumonia and pleurisy when re-solution and absorption commence.

Should a patient, not laboring under any febrile or inflammatory affection, be every now and then troubled with a pink deposit in the urine without any assignable cause, it will be found, in almost nine cases out of ten, that he is suffering from some chronic affection of the heart, liver, or spleen, with which is associated a tendency to gravel. In all such cases, therefore, steps should immediately be taken to counteract this disposition by the administration of alkaline tonics. Should there, however, be any counter-indication to the correct alkaline treatment, those acid salts are to be employed which, during their passage through the body, are converted into alkaline carbonates—such, for example, as citrates, tartrates, lactates, and acetatates. Every now and then an exceptional case may arise, where a mineral acid tonic is demanded; under such circumstances the above rule may be departed from, and the case treated according to its special requirements.

It not unfrequently happens that there are crystals of free uric acid scattered among an amorphous desposit of urates. This is more frequently the case in the course of chronic than of acute affections, and generally arises from there being an excess of acid in the urine, which has combined with some of the alkaline base and set a portion of the uric acid free.

The crystals of uric acid form on the bottom and sides of the vessel as the urine cools, and, if large they may be readily detected by the naked eye, in consequence of their yellow, red, or brown color. Uric acid, like urea, when crystalized in urine always takes up a part of the urohæmatin or any other coloring matter that may chance to be present. So that, the paler the urine, the less colored are the crystals; the darker the urine, the more colored the crystals. In abnormal urines containing blue, black, or saffron-colored pigment, the uric acid crystals are blue, black, or yellow; as in these variously-colored specimens now before you. The crystals are easily recognized by the naked eye, if the urine be put in a wine-glass and the deposit allowed to fall to the bottom.

Uric acid is not necessarily in excess when it crystalizes spontaneously—though that is the general rule. It has just been said that an excess of free acid induces its crystalization. I may now add that it is also deposited when from any cause the proportion of alkaline base is abnormally diminished. This arises from the circumstance that uric acid is much less soluble than any of its salts, even the urate of ammonia included.

The daily amount of uric acid excreted varies considerably in disease. It is generally in excess during the course of all fevers (yellow fever excepted), exanthematous affections, and inflammatory diseases. It has been found doubled in typhus (Parkes), greatly increased in small-pox, as well as considerably augmented in pneumonia. In certain diseases crystals of free uric acid are rpontaneously deposited in the urine. This is more particularly the case in hepatic, cardiac, and splenic diseases.

In liver affections there is frequently a spontaneous deposit of free uric acid among the amorphous urates, which, as already said, are so common in those affections. This is more particularly observable in cancer of that organ, in which case, too, the uric acid is generally in excess;—a fact frequently made use of in diagnosis, for in non-malignant hepatic disease especially towards its latter stages, the uric acid is found to be remarkably diminished.—*Med. Times and Gazette, May* 28, 1864, *p.* 584.

